# Schizophrenia and Polycythemia Vera: A Case Report

**DOI:** 10.1192/j.eurpsy.2024.331

**Published:** 2024-08-27

**Authors:** N. El Moussaoui, S. Bahetta, I. Belabbes, F. Laboudi, A. Ouanass

**Affiliations:** Arrazi University Psychiatric Hospital, Salé, Morocco

## Abstract

**Introduction:**

Schizophrenia is a severe mental disorder marked by abnormal interpretations of reality, often resulting in hallucinations, delusions, and disordered thinking that significantly impairs daily functioning and can be disabling. Lifelong treatment is necessary, and early intervention can help manage symptoms and improve long-term outcomes.

Polycythemia Vera (PV) is a chronic myeloproliferative neoplasm causing an excess of red blood cells in the peripheral blood (polyglobulia). While the disease typically presents with symptoms, it can also be asymptomatic and discovered incidentally during routine laboratory tests, leading to a diagnosis of polycythemia when no secondary cause is apparent.

While early 20th-century literature linked PV to intense neurological and psychiatric symptoms, contemporary studies rarely make such references.

**Objectives:**

The aim of this study is to explore, through a clinical case of a patient undergoing treatment for treatment-resistant schizophrenia with clozapine, and concurrently diagnosed with Polycythemia Vera, the potential causes of this condition. We seek to discern whether it represents mere comorbidities or if Polycythemia Vera is an adverse effect of antipsychotic treatment, particularly with clozapine.

**Methods:**

A 41-year-old patient, with a history of cranial trauma at the age of 5 and 19 years of treatment for schizophrenia, also has a tobacco use disorder. While hospitalized for the management of symptomatic reactivation of schizophrenia, despite being on clozapine, the patient underwent various therapeutic combinations with no observed clinical improvement. A few months later, follow-up blood tests indicated an elevation in all blood cell lines.

An internal medicine consultation was sought, resulting in the diagnosis of Polycythemia Vera.

**Results:**

The evaluations conducted led us to the conclusion that there are two distinct nosological entities, with the treatment of the psychiatric condition revealing true polycythemia. Even after reducing the doses of clozapine and changing the atypical antipsychotic, all subsequent evaluations showed no effectiveness in managing the psychiatric disorder or improvement in the hematological condition.

**Conclusions:**

In summary, schizophrenia is a severe and lifelong mental disorder requiring early intervention for symptom management. Polycythemia Vera (PV), a myeloproliferative disorder, typically presents with symptoms but can also be asymptomatic.

While early literature linked PV to intense neurological and psychiatric symptoms, contemporary studies seldom reference such associations. The coexistence of schizophrenia and PV in a patient underscores the need for comprehensive and interdisciplinary care to address the complex interplay between mental and physical health. Further research is needed to deepen our understanding of concurrent psychiatric and hematological conditions.

**Disclosure of Interest:**

None Declared

